# Supplementary figures and images for: O-GlcNAc glycosylation orchestrates fate decision and niche function of bone marrow stromal progenitors
Source: eLife. 2023 Mar 2;12:e85464. doi: 10.7554/eLife.85464 (PMC10032655; doi:10.7554/eLife.85464)

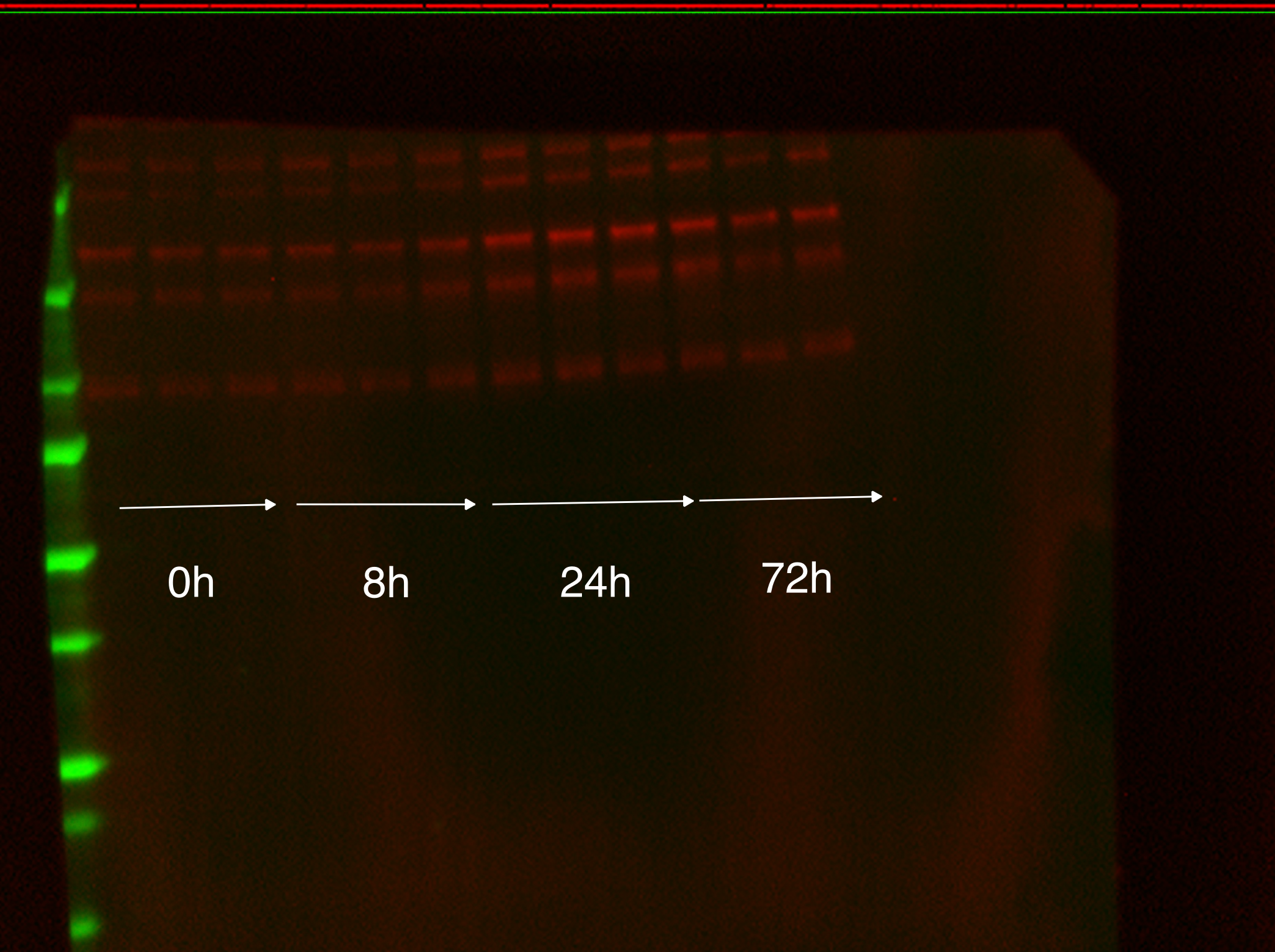

Supplement: Figure 2—source data 1. [file elife-85464-fig2-data1.zip › Figure 2source data 1/Fig.2D-O-GlcNAc.tif]

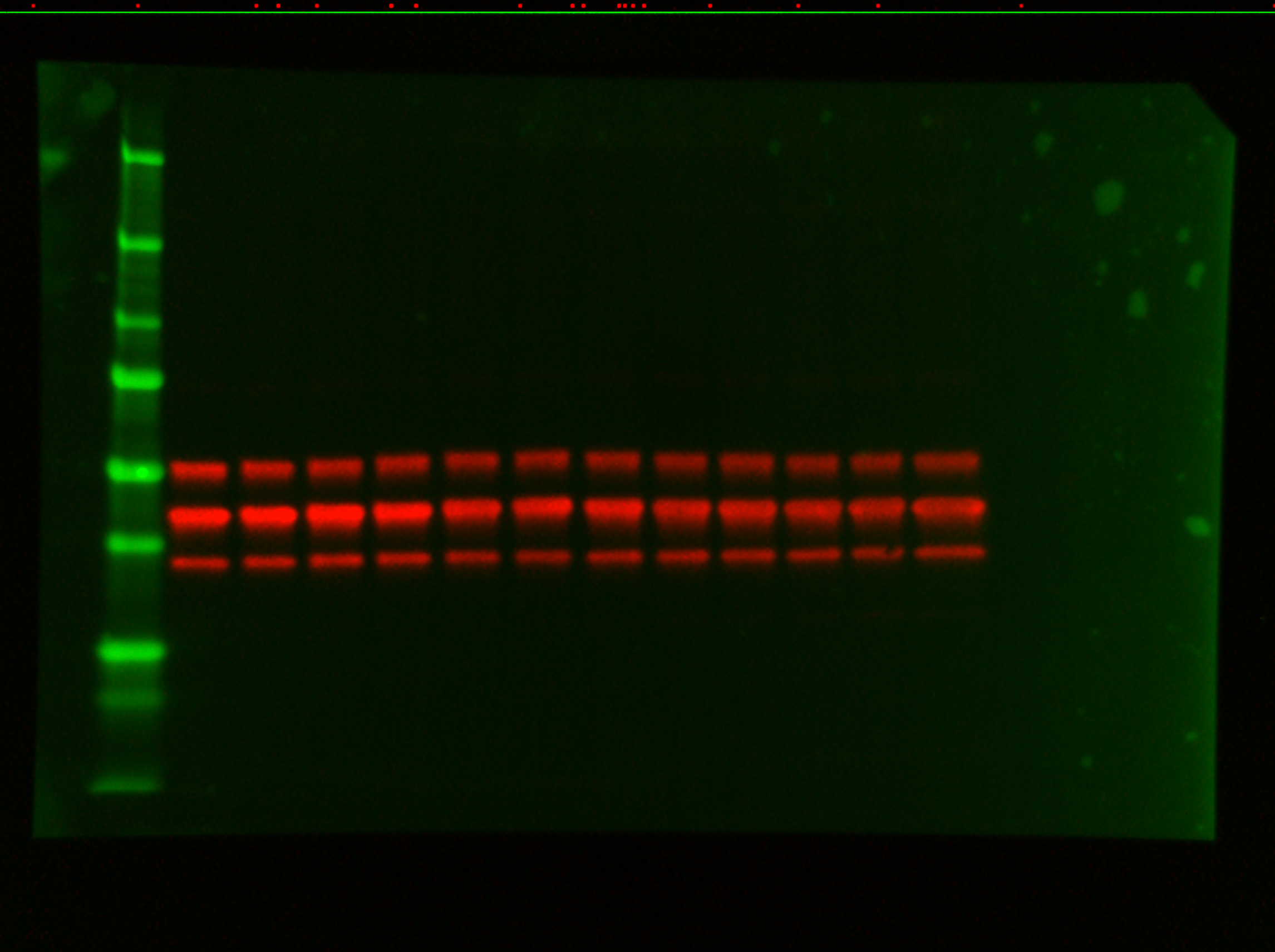

Supplement: Figure 2—source data 1. [file elife-85464-fig2-data1.zip › Figure 2source data 1/Fig.2D-loading.tif]

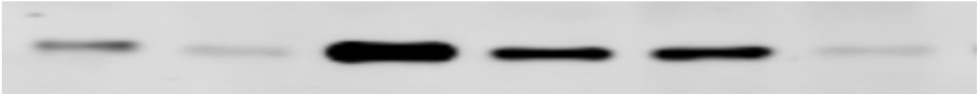

Supplement: Figure 2—source data 2. [file elife-85464-fig2-data2.zip › Fig.2G-O-GlcNAc.jpg]

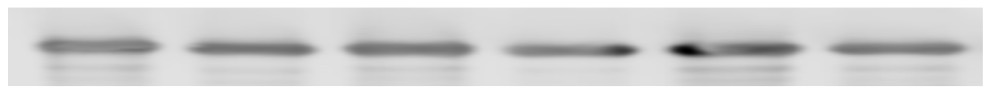

Supplement: Figure 2—source data 2. [file elife-85464-fig2-data2.zip › Fig.2G-RUNX2.jpg]

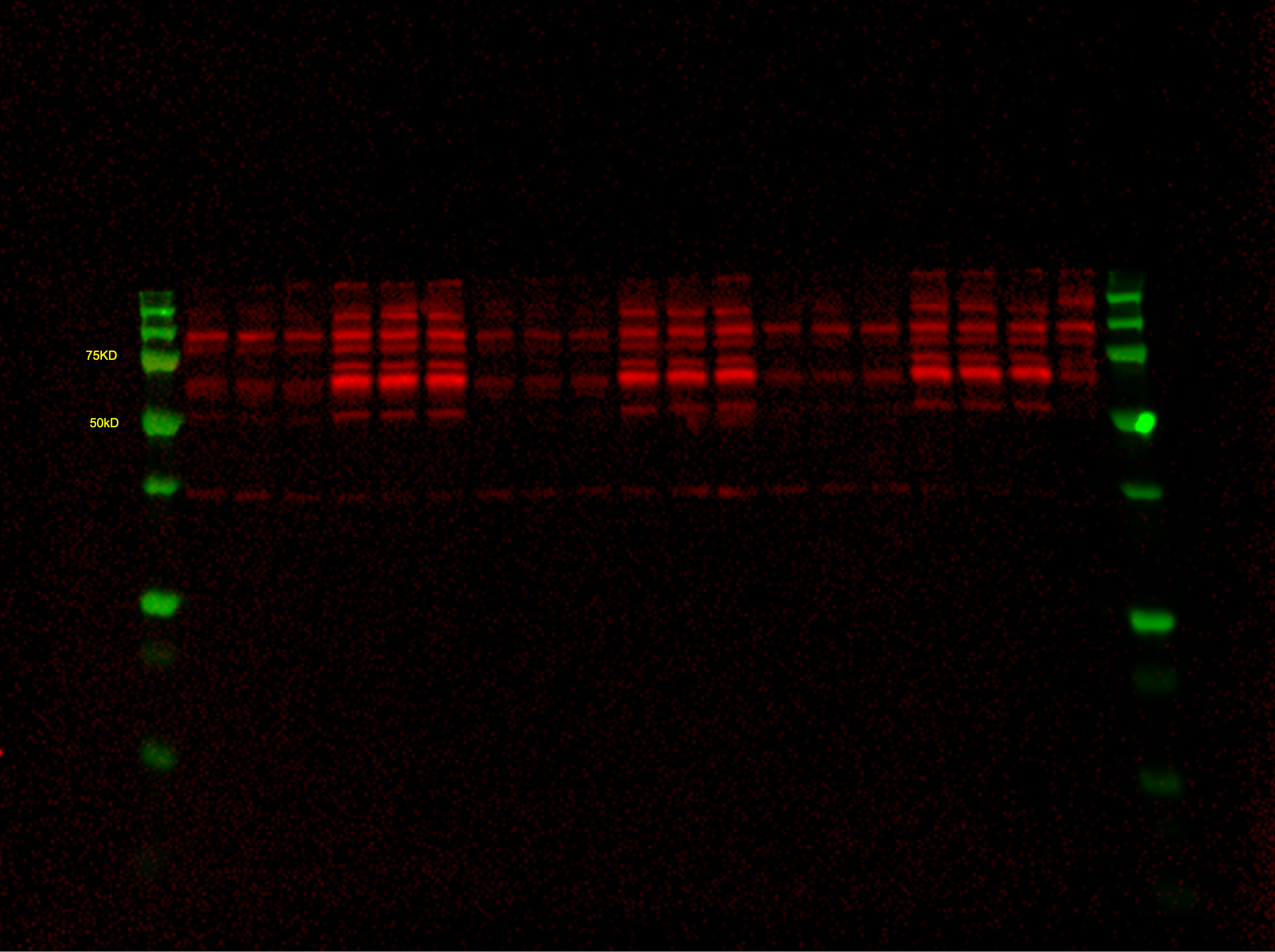

Supplement: Figure 4—source data 2. [file elife-85464-fig4-data2.zip › Fig.4G-O-GlcNAc.tif]

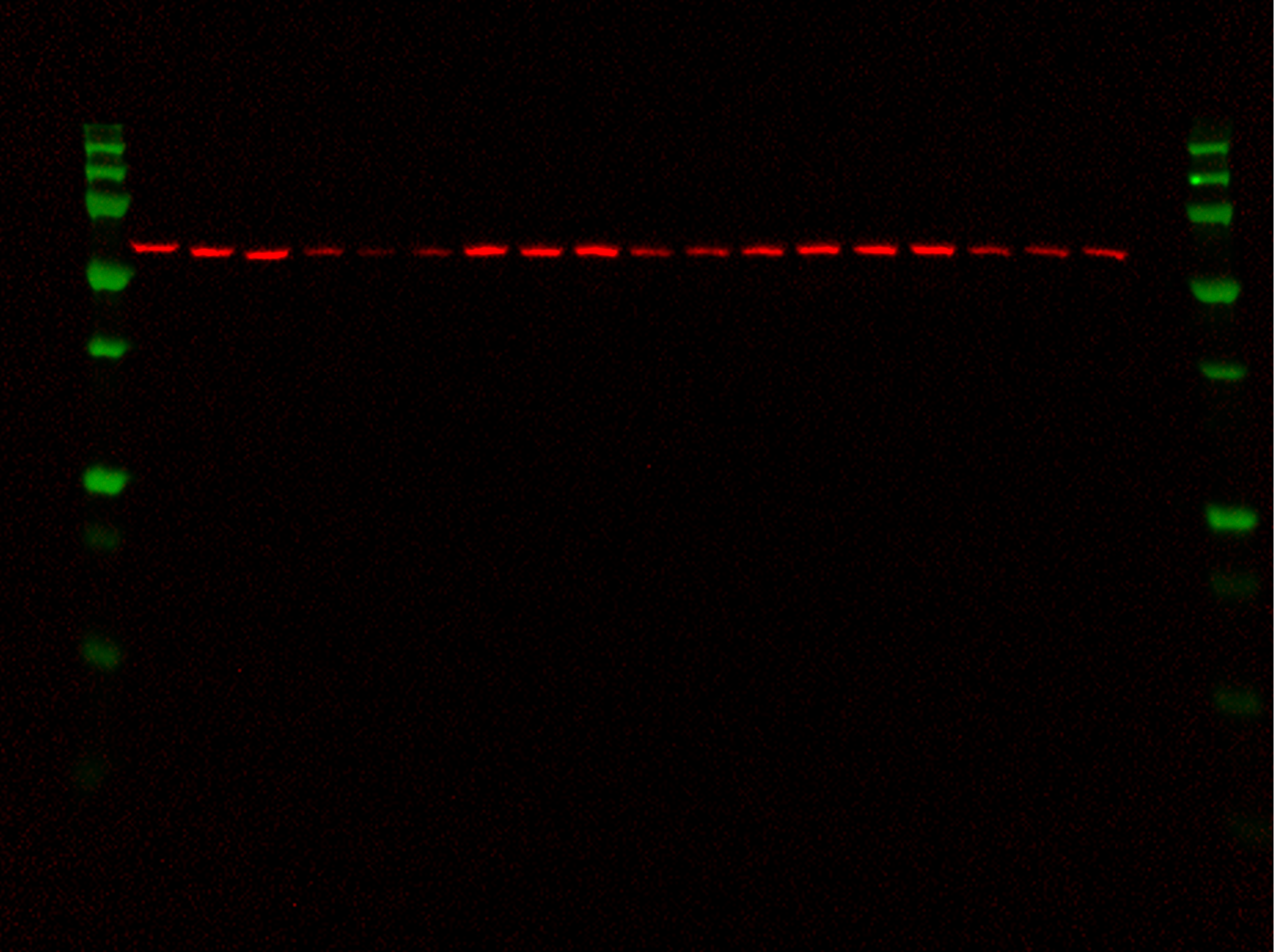

Supplement: Figure 4—source data 2. [file elife-85464-fig4-data2.zip › Fig.4G-Perilipin.tif]

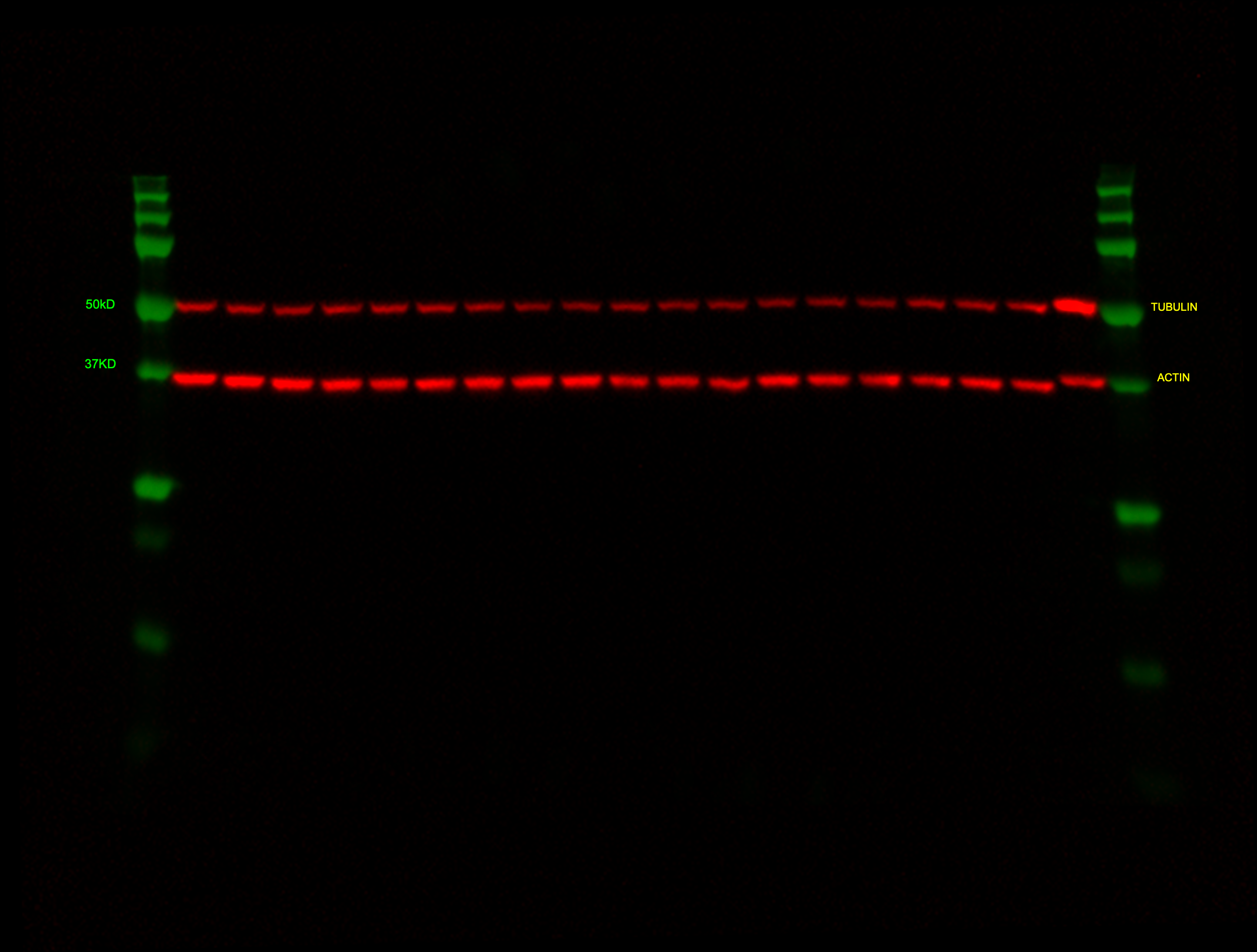

Supplement: Figure 4—source data 2. [file elife-85464-fig4-data2.zip › Fig.4G-Tubulin.tif]

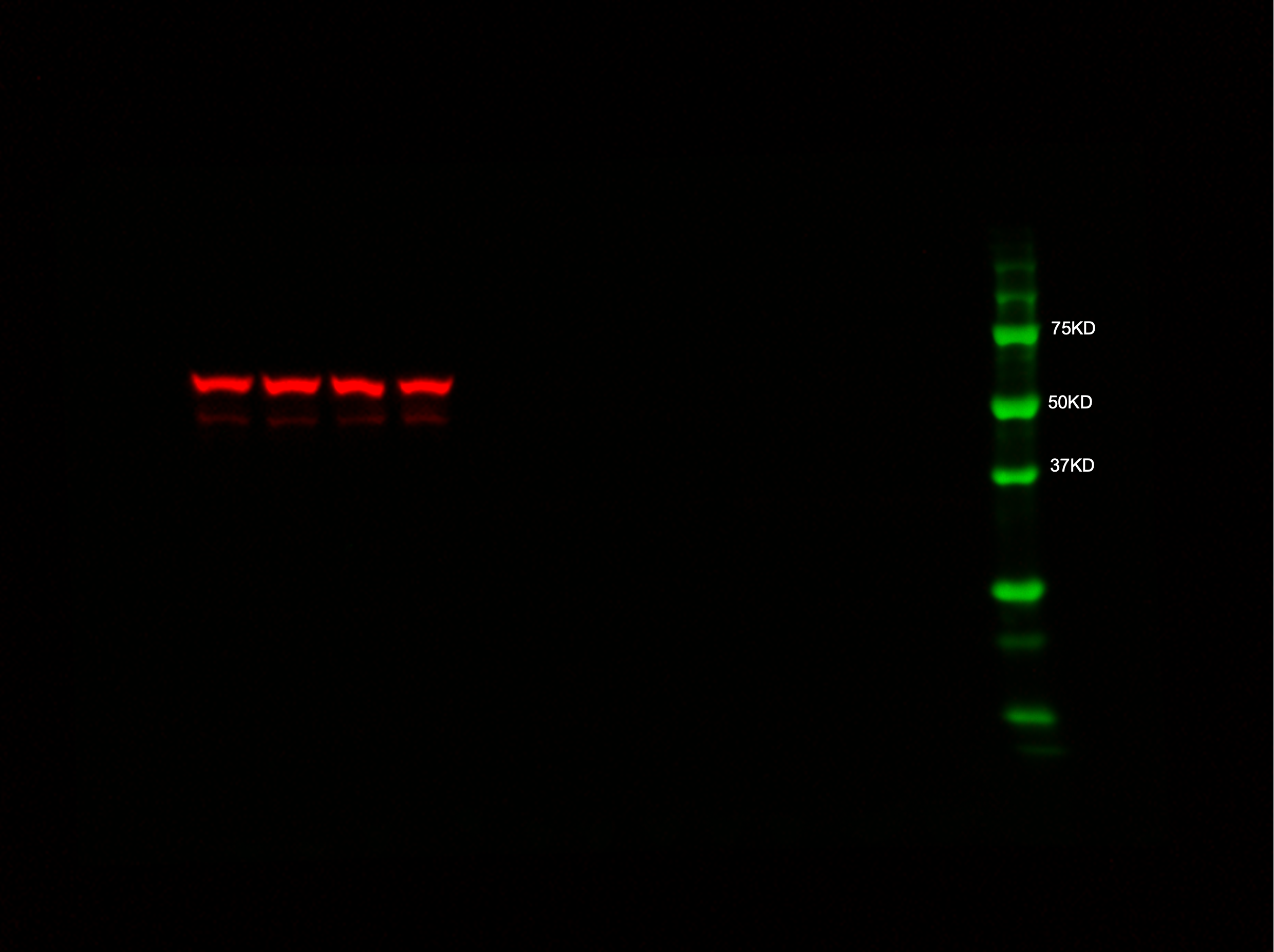

Supplement: Figure 4—figure supplement 1—source data 1. [file elife-85464-fig4-figsupp1-data1.zip › Fig.S4B-Perilipin.tif]

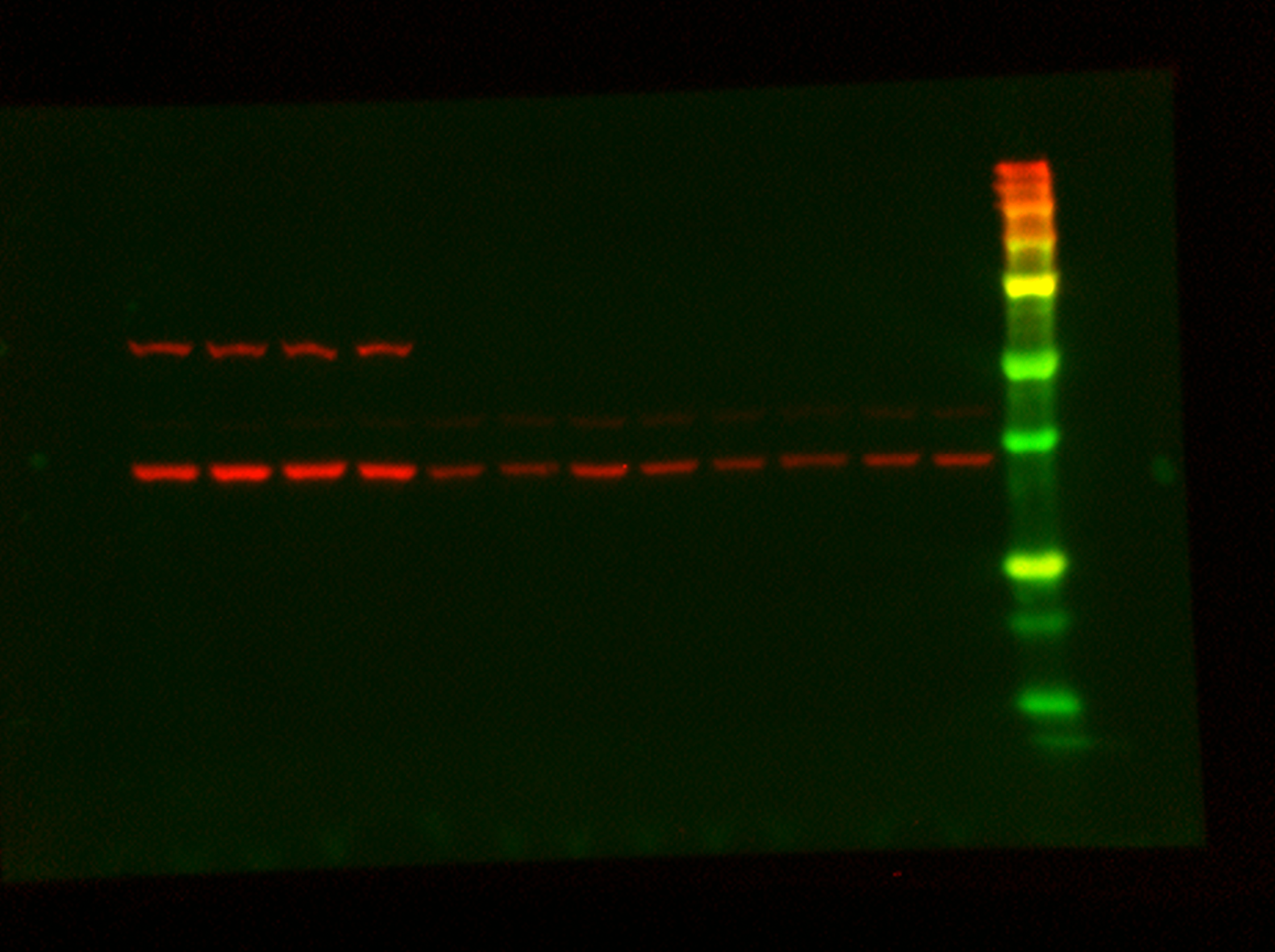

Supplement: Figure 4—figure supplement 1—source data 1. [file elife-85464-fig4-figsupp1-data1.zip › Fig.S4B-GAPDH.tif]

## Slide 1
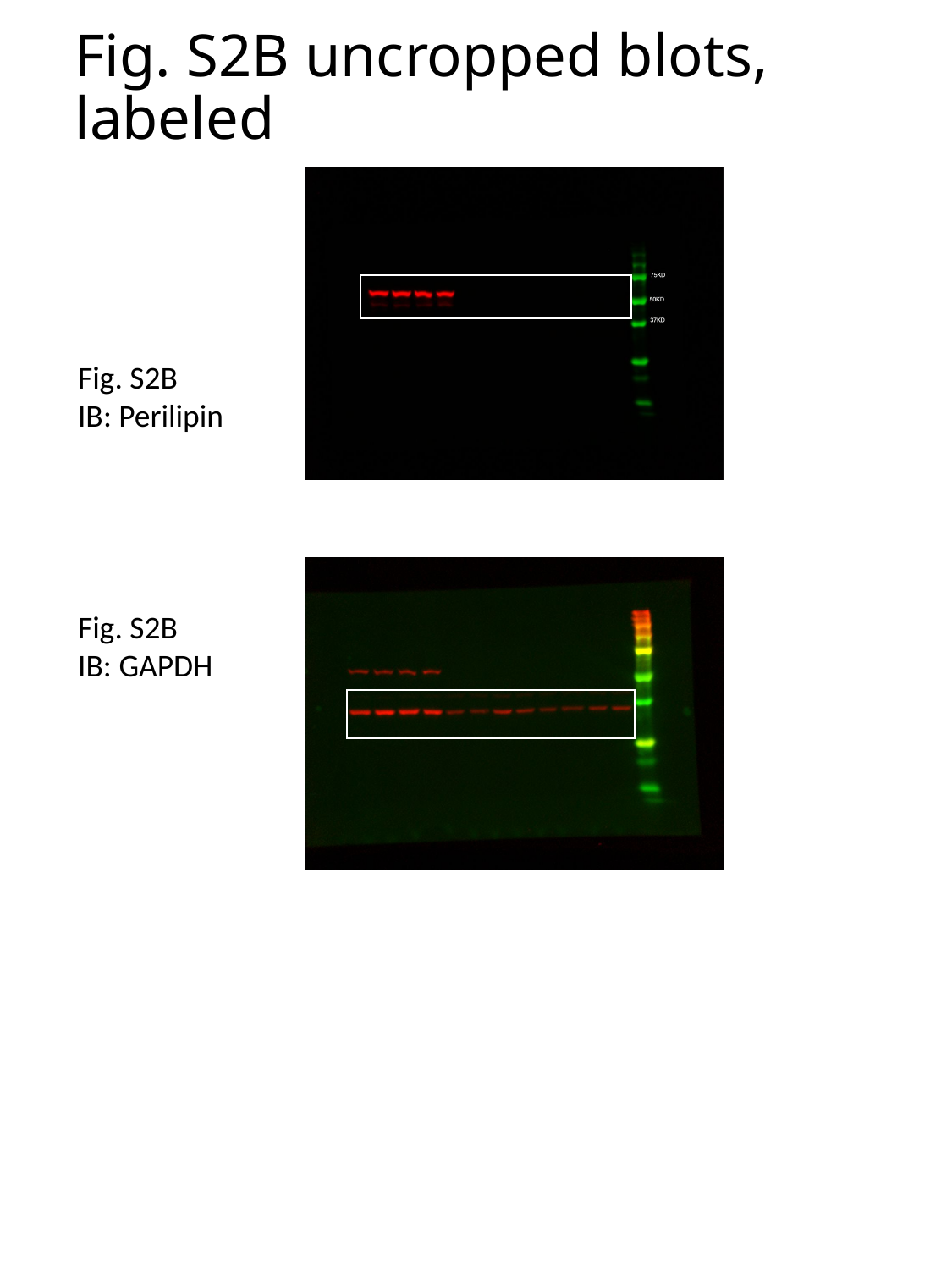

# Fig. S2B uncropped blots, labeled
Fig. S2B
IB: Perilipin
Fig. S2B
IB: GAPDH

Supplement: Figure 4—figure supplement 1—source data 1. [file elife-85464-fig4-figsupp1-data1.zip › Fig.S4B blots.pptx]
