# Supplementary material for: O-GlcNAc glycosylation orchestrates fate decision and niche function of bone marrow stromal progenitors
Source: eLife. 2023 Mar 2;12:e85464. doi: 10.7554/eLife.85464 (PMC10032655; doi:10.7554/eLife.85464)
Supplement: Figure 2—source data 2. [file elife-85464-fig2-data2.zip › Fig.2G blots.pptx]

## Slide 1
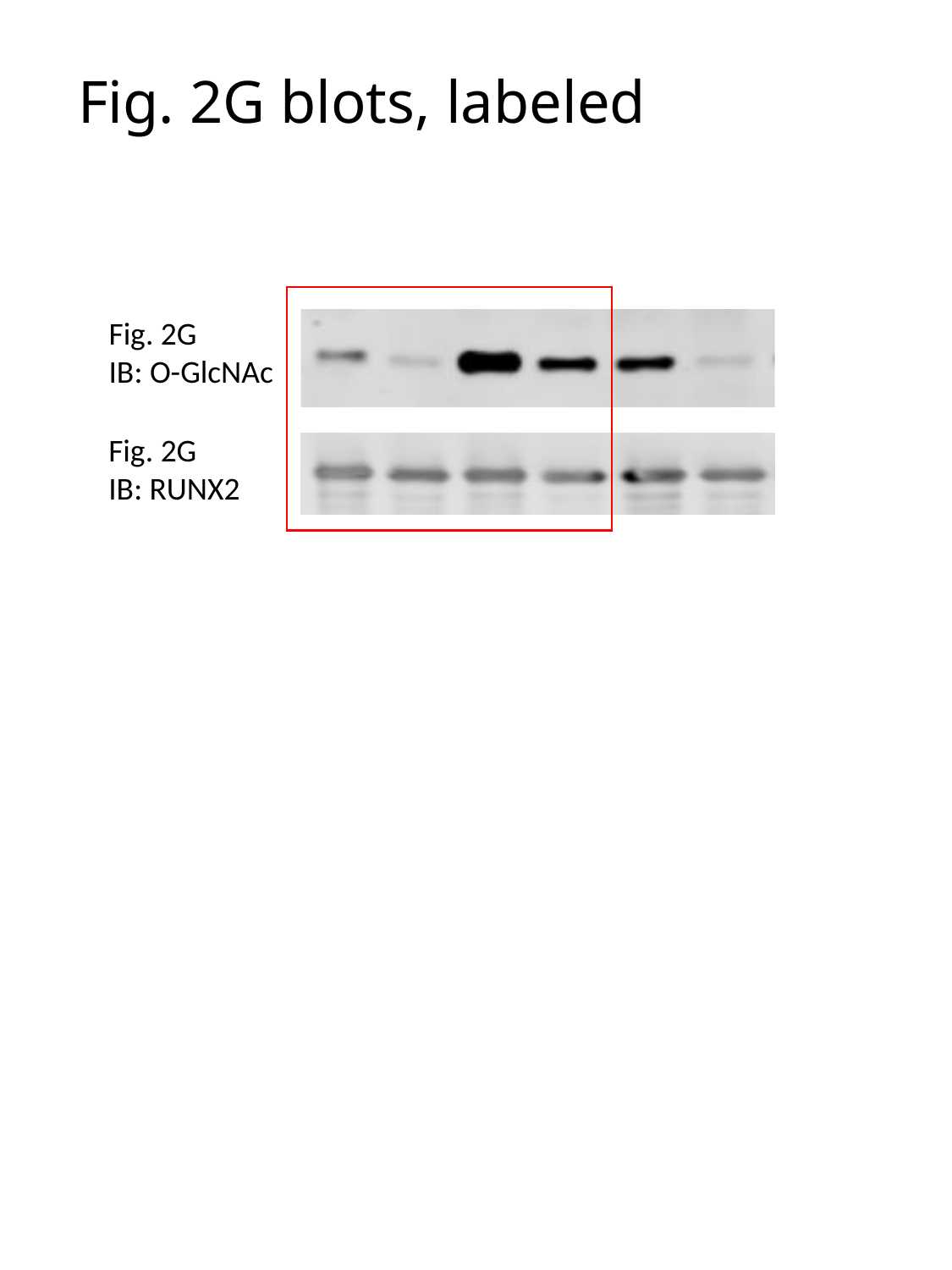

# Fig. 2G blots, labeled
Fig. 2G
IB: O-GlcNAc
Fig. 2G
IB: RUNX2
Effect of glucose concentration on O-GlcNAcylation of wt and GlcNAc-deficient (S32A/S33A/S371A) Runx2.
S32A/S33A/S371A
S32A/S33A/S371A
S32A/S33A/S371A
WT Runx2
WT Runx2
WT Runx2
WB: O-GlcNAc
WB: Runx2
Thiamet G - - + + - -
Glucose (mM) 5 5 5 5 25 25
IP: Flag-Runx2
Effect of glucose concentration on O-GlcNAcylation of wt and GlcNAc-deficient (S32A/S33A/S371A) Runx2.
S32A/S33A/S371A
S32A/S33A/S371A
S32A/S33A/S371A
WT Runx2
WT Runx2
WT Runx2
WB: O-GlcNAc
WB: Runx2
Thiamet G - - + + - -
Glucose (mM) 5 5 5 5 25 25
IP: Flag-Runx2
